# Metabolomics, antioxidant, and enzyme inhibitory effects of *Citrus aurantium* fruits

**DOI:** 10.3389/fchem.2025.1613827

**Published:** 2025-06-30

**Authors:** Omayma A. Eldahshan, Salwa Bouabdallah, Rawan M. Abd El-khalek, Mahmoud A. El Hassab, Gokhan Zengin, Ahmed T. Negmeldin, Eman F. Khaleel, Wagdy M. Eldehna, Nada M. Mostafa

**Affiliations:** ^1^ Department of Pharmacognosy, Faculty of Pharmacy, Ain Shams University, Cairo, Egypt; ^2^ Center of Drug Discovery, Research and Development, Faculty of Pharmacy, Ain Shams University, Cairo, Egypt; ^3^ Environmental Biomonitoring Laboratory LBE (LR01/ES14), Faculty of Sciences Bizerta, Carthage University, Zarzouna, Tunisia; ^4^ Faculty of Medicine, New Giza University, Giza, Egypt; ^5^ Department of Medicinal Chemistry, Faculty of Pharmacy, King Salman International University (KSIU), South Sinai, Egypt; ^6^ Department of Biology, Science Faculty, Selcuk University, Konya, Türkiye; ^7^ Department of Pharmaceutical Sciences, College of Pharmacy and Thumbay Research Institute for Precision Medicine, Gulf Medical University, Ajman, United Arab Emirates; ^8^ Department of Pharmaceutical Organic Chemistry, Faculty of Pharmacy, Cairo University, Cairo, Egypt; ^9^ Department of Medical Physiology, College of Medicine, King Khalid University, Asir, Saudi Arabia; ^10^ Department of Pharmaceutical Chemistry, Faculty of Pharmacy, Kafrelsheikh University, Kafrelsheikh, Egypt

**Keywords:** Citrus aurantium, bitter orange, Rutaceae, GC-MS, antioxidant, enzyme inhibition

## Abstract

**Introduction:**

The genus *Citrus* comprises a large number of economically important fruit crops. They are recognized globally as functional foods and in the food, pharmaceutical, and cosmetic industries.

**Methods:**

We present herein the chemical composition of the hexane extracts of *Citrus aurantium* (bitter orange) fruits and leaves by GC-MS for the first time, in addition to their antioxidant and enzyme inhibitory activities *in vitro*.

**Results and Discussion:**

GC-MS revealed nootkatone (15.29%), decyl anthranilate (11.58%), neryl acetate (7.83%), and linalool acetate (6.83%) as major components of fruit extract; while the leaves extract contained mainly lupeol (24.32%), linalool (16.47%), friedelan-3-one (16.40%) and linalool acetate (12.31%). The extracts showed potential inhibitory activities against acetylcholinesterase (AChE), butyrylcholinesterase (BChE), tyrosinase, amylase, and glucosidase enzymes. Results were confirmed by in silico molecular docking studies on the respective enzymes' active sites, viz NADPH oxidase, BChE, tyrosinase, α-amylase, and α-glucosidase. Amongst the docked compounds, lupeol showed the best binding affinities to NADPH oxidase, butyrylcholinesterase BChE, and α- glucosidase; while linalool acetate and neryl acetate showed the best activities against tyrosinase and α-amylase enzymes, respectively. In conclusion, bitter orange waste products can be a potentially important source of antioxidants and useful supplements.

## 1 Introduction

The genus *Citrus* belongs to the family Rutaceae, and comprises various species of diverse sizes and forms, such as *Citrus sinensis* (orange), *Citrus reticula* (mandarine), *Citrus limon* (lemon), *Citrus paradise* (grapefruit), and *Citrus junos* (yuzu) (Ashmawy et al., 2024). *Citrus* plants are economically important fruit crops. Annual production of *Citrus* plants has accomplished more than 126 million tons (FAO, 2017), whereas, 25 million of which were produced in the Mediterranean region. Plants of the genus *Citrus* are recognized for their fruits, juices, and also as functional foods. In addition, *Citrus* varieties are used in the food industry and their essential oils are extensively used in the pharmaceutical and cosmetic industries (Bouabdallah et al., 2022; De Pasquale et al., 2006). *Citrus* species are rich in essential oils, vitamins B9, E, and C, antioxidants, dietary fiber, and coumarins with important health-promoting properties (Hwang et al., 2012; Othman et al., 2016; Ashmawy et al., 2019). Despite, the chemical composition of *Citrus* essential oils has been extensively studied in various studies, differences in their phytoconstituents were observed due to the differences in the species/cultivars, climate, origin, season, ripening stage, extraction, and analytical methods used (Bouabdallah et al., 2022; De Pasquale et al., 2006; Bora et al., 2020).

*Citrus aurantium L* (*Citrus aurantium*) commonly named bigarade, sour, or bitter orange, is an enduring tree that can extend up to 5 m tall, characterized by its white flowers. It is native to Africa and Syria but was cultivated in Spain, the United States, and Italy (Bora et al., 2020; Hosni et al., 2010). The chemical composition of the essential oil of *C. aurantium* (bitter orange) was evaluated in various plant parts during different seasons (Elshafie, 2022; Djenane, 2015). Many studies were focused on oils from *C. aurantium* peels and limonene was found to be the major component (Hosni et al., 2010; Elshafie, 2022; Djenane, 2015; Radan et al., 2018; Sarrou et al., 2013; Mohagheghniapoura et al., 2018; Sanei-Dehkordi et al., 2016; Azanchi et al., 2014; Khodabakhsh et al., 2015; Hsouna et al., 2013). However, the volatile constituents from leaves and fruits have not received much attention in the literature, except for a few studies (Radan et al., 2018; Hsouna et al., 2013) that studied the leaves and showed that linalool was the main essential oil component. *C. aurantium* essential oils have been reported for their antioxidant, anti-inflammatory, insecticidal antibacterial, and antifungal activities (Radan et al., 2018; Hsouna et al., 2013; Bnina et al., 2019; Ben Hsouna et al., 2019).

To the best of our knowledge, there are no reports concerning the fruit rind volatiles or the *n*-hexane extract from leaves and fruits of *C. aurantium*. Thus, the main objective of the current study is to explore the composition of *n*-hexane leaves and fruit extracts of *C. aurantium* and to evaluate their antioxidant and enzyme inhibitory potentials against acetylcholinesterase (AChE), butyrylcholinesterase (BChE), tyrosinase, amylase, and glucosidase enzymes. Molecular docking studies were also carried out on the major components of each extract to confirm the observed results.

## 2 Materials and methods

### 2.1 Plant material and extraction

#### 2.1.1 Preparation of the *n*-hexane extract

The fresh leaves and fruit peels of *Citrus aurantium* (100 g) were obtained from a private farm in Menoufia, Egypt. The Voucher specimen was kept at the herbarium of the Pharmacognosy Department, Ain Shams University, Egypt (code: PHG-P-CA-461). The fresh leaves and fruits parts were extracted with *n*-hexane three times separately. The filtrate was completely evaporated *in vacuo* at 40°C until dryness to obtain the dried residue of the *n*-hexane extract (2.4, 2.2 g). Both extracts were stored separately in a refrigerator for further analysis.

### 2.2 Gas chromatography/mass spectrometry (GC-MS)

The GC-MS of the *n*-hexane extracts was accomplished using a Shimadzu GCMS-QP equipment with a TRACE GC Ultra Gas Chromatograph (THERMO Scientific Corp., United States), conjugated with a thermo-mass detector at the Pharmacognosy Department, Ain Shams University, Cairo, Egypt. The GC-MS had a TG-5MS capillary column (30 m × 0.25 mm i. d., 0.25 μm film thickness) (Restek, United States). The capillary column was directly coupled to a quadrupole mass spectrometer (SSQ 7000; Thermo-Finnigan). Analysis of a diluted sample (1% v/v; injected volume = 1 µL) was carried out using helium as carrier gas at a constant flow rate of 1.0 mL/min and a split ratio of 1:15. The oven temperature was adjusted at 80°C for 2 min (isothermal), then raised 5.0 °C/min to reach 300°C (programmed) and held for 5 min (isothermal). The injector and detector temperature were held at 280°C. The mass spectra were obtained by adjusting the following parameters as follow: interface temperature = 280°C, ion source temperature = 200°C, and electron ionization (EI) mode = 70 eV, using a scan spectral range at *m/z* 35–500. The relative proportions of the *n-*hexane extract constituents were expressed as percentages obtained by peak area normalization.

### 2.3 GC-MS identification of chemical components of the *n-*hexane extracts

The components of the *n-*hexane extracts were tentatively considered by matching their GC-MS spectra, fragmentation patterns, mass numbers, and Kovats retention indices with those published in the Wiley, NIST library and literature reports (Taha and Eldahshan, 2017; Abd El-Ghffar et al., 2017; Shahat et al., 2017; Azab et al., 2017; Eldahshan and Halim, 2016). The retention indices were calculated relative to a homologous series of *n*-alkanes (C_8_-C_28_) injected under the same conditions. The peak area percent of each compound relative to the area percent of the entire FID chromatogram (100%) was calculated.

### 2.4 Antioxidant assays

Antioxidant assays were carried out according to previously reported methodologies (Zengin et al., 2023a; Zengin et al., 2023b). The antioxidant potential was expressed as mg Trolox equivalents (TE)/g extract in 2,2-diphenyl-1-picrylhydrazyl (DPPH) and 2,20 -azino-bis (3-ethylbenzothiazoline6-sulfonic acid) (ABTS) radical scavenging, cupric reducing antioxidant capacity (CUPRAC), and ferric reducing antioxidant power (FRAP) tests, mmol TE/g extract in phosphomolybdenum assay (PBD), and mg ethylenediaminetetraacetic acid equivalents (EDTAE)/g extract in metal chelating assays (MCA).

For DPPH activity, 1 mL of sample (1 mg/mL) was added to 4 mL of DPPH dissolved in methanol (0.004%). Then after 30 min in dark, the absorbance was read at 517 nm.

ABTS activity was assessed for which 1 mL of sample (1 mg/mL) was added to 2 mL of ABTS solution. The sample absorbance was measured at 734 nm after 30 min at room temperature.

For CUPRAC, 0.5 mL of sample (1 mg/mL) was added to 3 mL of CUPRAC reaction mixture and the absorbance was read at 450 nm after incubation at room temperature for 30 min.

For FRAP, 0.1 mL of sample (1 mg/mL) was added to 2 mL FRAP reagent and absorbance was read at 593 nm after a 30 min incubation at room temperature.

Regarding phosphomolybdenum, 0.3 mL of sample (1 mg/mL) was addedd to 3 mL of reagent and after 90 min at 95°C, the absorbance was read at 695 nm.

Considering the metal chelating activity, 2 mL of sample (1 mg/mL) was added to 0.05 mL of ferrous chloride solution (2 mM) with adding 0.2 mL of 5 mM ferrozine then absorbance was read at room temperature at 562 nm after 10 min.

### 2.5 Enzyme inhibitory assays

The enzyme inhibitory assays were carried out according to previously reported methodologies (Zengin et al., 2023a; Zengin et al., 2023b). The acetylcholinesterase (AChE) and butyrylcholinesterase (BChE) inhibition were expressed as mg galanthamine equivalents (GALAE)/g extract; tyrosinase inhibition was expressed as mg kojic acid equivalents KAE/g extract; amylase and glucosidase inhibition were expressed as mmol acarbose equivalents (ACAE)/g extract.

For each of AChE or BChE inhibitory activities, 50 μL of sample solution (1 mg/mL); was mixed with 125 μL DTNB (5,5-dithio-bis(2-nitrobenzoic) acid, Sigma, St. Louis, MO, United States) and 25 μL AChE or BChE in Tris–HCl buffer (pH 8.0) incubated at 25°C for 15 min. The reaction was initiated with 25 μL of acetylthiocholine iodide (ATCI) or butyrylthiocholine chloride (BTCl), a blank is done and the absorbance of the blank was subtracted from sample.

25 μL of sample (1 mg/mL) was added to 40 μL tyrosinase solution and 100 μL phosphate buffer (pH 6.8) for determination of tyrosinase activity after 15 min at 25°C. The reaction was initiated with 40 μL L-DOPA. A blank is done without tyrosinase solution. The absorbance at 492 nm of blank was subtracted from sample.

For α-amylase activity, 25 μL of sample (1 mg/mL) was added to 50 μL α-amylase solution in phosphate buffer (pH 6.9) after which it was incubated for 10 min at 37°C. The reaction was initiated by adding 50 μL starch solution and leave 10 min at 37°C. The reaction was stopped by 1 M HCl (25 μL). Then add 100 μL iodine-potassium iodide solution. nm. The absorbance of blank was subtracted from sample at 630.

For α-glucosidase activity, 50 μL of sample (1 mg/mL) was added to equal volumes of each of glutathione and α-glucosidase solution in phosphate buffer (pH 6.8) and PNPG (4-N-trophenyl-α-D-glucopyranoside, Sigma) and incubated for 15 min at 37°C. Ablank was done without the enzyme and reaction was stopped by 50 μL sodium carbonate (0.2 M). The absorbance at 400 nm of blank was subtracted from that of the sample.

### 2.6 Docking study

The X-ray 3D structures of NADPH oxidase, butyrylcholinesterase, tyrosinase, α-amylase, and α-glucosidase were downloaded from the protein data bank using the following IDs: 2cdu, 6esj, 5m8q, 4gqq and 3wy2, respectively. Vina autodock and MGL tools were employed to conduct the docking studies (Trott and Olson, 2010; El Hassab et al., 2021). The major compounds identified in the *n*-hexane fruit and leaves extract of *C. aurantium* were implemented in the docking study. All five receptors and the six compounds were saved in a pdbqt format using MGL tools as an essential requisite by Vina autodock. The active site of each target was determined from the binding of the corresponding co-crystalized ligand. Finally, the docking results were inspected by the Discovery Studio visualizer which was also used to generate the 2D interaction diagrams (Shady et al., 2022; El-Nashar et al., 2023).

## 3 Results

### 3.1 GC-MS of *C. aurantium* fruit extract

**FIGURE 1:**
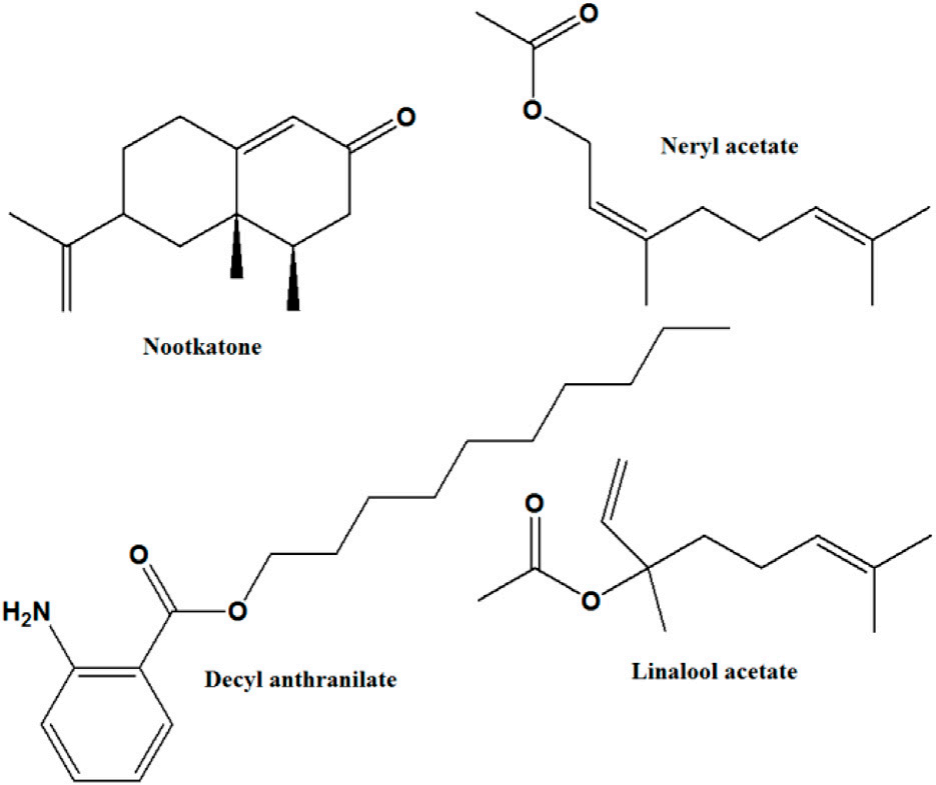
Structures of major compounds identified in *n*-hexane fruit extract of *Citrus aurantium*.

Twenty-five compounds were tentatively identified from the *n*-hexane fruit extract of *C. aurantium,* representing about 90.59% of the total peak area, and are presented in Table 1. Major identified compounds are shown in Figure 1.

**TABLE 1:** The chemical composition of the *n-*hexane fruit extract of *C. aurantium was* tentatively identified by GC-MS.

No.	Retention Time	Compounds	Molecular Formula	RI *Cal*	RI *Rep*	Method of identification	% Sample
1	9.782	Limonene	C_10_H_16_	1,018	1,024	MS, KI	3.47
2	12.060	Linalool	C_10_H_18_O	1,082	1,088	MS, KI	1.40
3	14.895	Terpineol	C_10_H_18_O	1,143	1,143	MS, KI	2.70
**4**	**16.814**	Linalool acetate	**C**_**12**_ **H**_**20**_ **O**_**2**_	**1,272**	**1,272**	MS, KI	**6.83**
5	19.932	Lavandulyl acetate	C_12_H_20_O_2_	1,270	1,288	MS, KI	1.03
**6**	**20.453**	Neryl-acetate	**C**_**14**_ **H**_**24**_ **O**_**2**_	**1,352**	**1,359**	MS, KI	**7.83**
7	21.176	Geranyl-acetate	C_12_H_20_O_2_	1,380	1,379	MS, KI	1.00
8	21.490	Caryophyllene	C_15_H_24_	1,417	1,417	MS, KI	4.50
9	23.122	Germacrene D	C_15_H_24_	1,481	1,484	MS, KI	4.11
10	23.441	Guaia-1 (10),11-diene	C_15_H_24_	1,493	1,492	MS, KI	1.08
12	25.130	Nerolidol	C_15_H_26_O	1,564	1,561	MS, KI	3.43
**14**	**30.890**	Nootkatone	**C**_**15**_ **H**_**22**_ **O**	**1800**	**1806**	MS, KI	**15.29**
15	37.563	Osthole	C_15_H_16_O_3_	2,143	2,140	MS, KI	4.00
16	38.332	Citronellyl anthranilate	C_17_H_25_NO_2_	2,184	2,180	MS, KI	0.71
**17**	**39.402**	Decyl anthranilate	**C**_**17**_ **H**_**27**_ **NO**_**2**_	**2,244**	**2,241**	MS, KI	**11.58**
19	40.207	Tricosane	C_23_H_48_	2,307	2,300	MS, KI	1.54
20	43.545	Octacosane	C_28_H_58_	2,804	2,800	MS, KI	1.76
21	44.437	Totarolone	C_20_H_28_O_2_	2,545	2,542	MS, KI	2.79
22	45.688	sugiol	C_20_H_28_O	2,625	2,629	MS, KI	1.72
23	46.640	Heptacosane	C_27_H_56_	2,688	2,700	MS, KI	3.23
24	49.515	Nonacosane	C_29_H_60_	2,887	2,900	MS, KI	4.26
25	52.205	Hentriacontane	C_31_H_64_	3,086	3,100	MS, KI	6.33
%Total							90.59

RI, cal, Calculated retention Index on HP-5 MS, RI, rep: Reported retention index. Major compounds are in bold.

### 3.2 GC-MS of *C. aurantium* leaves extract

**FIGURE 2:**
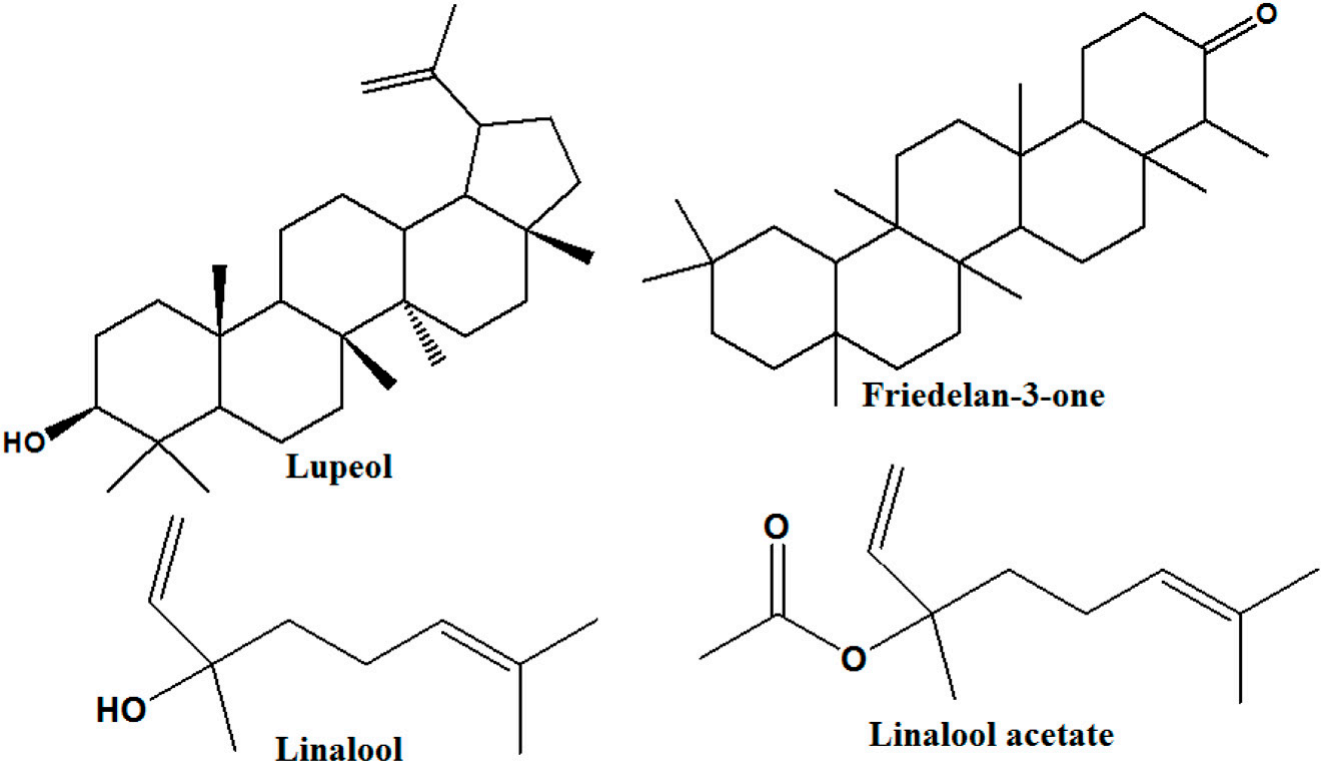
Structures of major compounds identified by *n*-hexane leaves extract of *Citrus aurantium*.

Thirteen compounds were tentatively identified from the *n*-hexane leaves extract of *C. aurantium,* representing about 90.34% of the total peak area, and are presented in Table 2. Major identified compounds are shown in Figure 2.

**TABLE 2:** Chemical composition of *n-*hexane leaves extract of *C. aurantium* tentatively identified by GC-MS.

No.	Ret. Time	Compounds	Molecular Formula	RI *Cal*	RI *Rep*	Method of identification	% Sample
1	6.584	Isocitronellene	C_10_H_18_	918	918	MS, KI	0.33
2	6.711	α-Thujene	C_10_H_16_	922	923	MS, KI	0.70
3	7.046	α-Pinene	C_10_H_16_	933	932	MS, KI	0.18
4	7.460	Camphene	C_10_H_16_	947	946	MS, KI	0.23
5	8.877	Octanol	C_18_H_18_O	995	994	MS, KI	0.20
**6**	**12.098**	Linalool	**C**_**10**_ **H**_**18**_ **O**	**1,098**	**1,095**	MS, KI	**16.47**
**7**	**16.834**	Linalool-acetate	**C**_**12**_ **H**_**20**_ **O**_**2**_	**1,253**	**1,254**	MS, KI	**12.31**
8	44.441	Totarolone	C_20_H_28_O_2_	2,545	2,542	MS, KI	0.23
9	49.526	Nonacosane	C_29_H_60_	2,888	2,900	MS, KI	1.95
**10**	**52.244**	Hentriacontane	**C**_**36**_ **H**_**74**_	**3,089**	**3,100**	MS, KI	**11.54**
11	54.964	Tritriacontane	C_36_H_74_	3,288	3,300	MS, KI	5.48
**12**	**57.423**	Lupeol	**C**_**30**_ **H**_**50**_ **O**	**3,510**	-	MS	**24.32**
**13**	**60.181**	Friedelan-3-one	**C**_**30**_ **H**_**50**_ **O**	**3,700**	-	MS	**16.40**
%Total							**90.34**

Linear retention index on HP-5 MS, column calculated according to the Van Den Dool and Kratz formula (1963). RI, cal: Calculated retention Index, RI, rep: Reported retention index. Major compounds are in bold.

### 3.3 Total bioactive components and antioxidant properties

In the present study, we determined the total content of phenolics and flavonoids in the tested extracts using spectrophotometric methods. As can be seen in Table 3, the leaf (13.61 mg GAE/g) and fruit extracts (13.24 mg GAE/g) contained almost part of the total phenol content. However, the total flavonoid content in leaf extract (6.31 mg RE/g) was higher than that of fruits (2.15 mg RE/g).

**TABLE 3:** Antioxidant effects of the *n*-hexane extracts.

Samples	TPC (mg GAE/g)	TFC (mg RE/g)	DPPH (mg TE/g)	ABTS (mg TE/g)	CUPRAC (mg TE/g)	FRAP (mg TE/g)	PBD (mmol TE/g)	MCA (mg EDTAE/g)
Leaves	13.61 ± 0.51	6.31 ± 0.44	13.96 ± 0.4	19.92 ± 2.66	50.91 ± 1.71	33.69 ± 1.08	3.13 ± 0.17	29.02 ± 0.51
Fruits	13.24 ± 0.45	2.15 ± 0.12	5.83 ± 0.50	13.44 ± 2.80	31.56 ± 0.66	26.21 ± 0.33	2.66 ± 0.10	22.53 ± 2.32

Values are reported as mean ± SD, of three parallel experiments. TE: trolox equivalent; EDTAE: EDTA, equivalent; na: not active. CUPRAC, cupric reducing antioxidant capacity; FRAP: ferric reducing antioxidant power; MCA: metal chelating ability assay; PBD, phosphomolybdenum assay. *: significantly different from the corresponding essential oil value at p < 0.05. using Independent Samples T-Test.

The antioxidant properties of n-hexane extracts were investigated through various chemical tests, including radical scavenger (DPPH and ABTS), reducing capacity (CUPRAC, FRAP and phosphomolybdenum) and metal chelation. The results are summarized in Table 3. In DPPH and ABTS assays, the leaf extract (DPPH: 13.96 mg TE/g; ABTS: 19.92 mg TE/g) showed a stronger ability than the fruit extract (DPPH: 5.83 mg TE/g; ABTS: 13.44 mg TE/g). Similar to tests on the radical scavenger, leaf extract was found to have the best reducing abilities compared to the fruit extract. In addition, the metal chelating capacity of leaves (29.02 mg EDTAE/g) was higher than that of fruits (22.53 mg EDTAE/g).

### 3.4 Enzyme inhibitory effects

The enzyme inhibitory effects of the tested extracts were investigated against several enzymes including cholinesterase (AChE and BChE), tyrosinase, amylase and glucosidase. The results are given in Table 4. In cholinesterase inhibition assays, the fruit extract (2.51 mg GALAE/g) exhibited more potent inhibitory effect on AChE than the leaves extract (1.63 mg GALAE/g); while the leaves extract (2.24 mg GALAE/g) displayed a higher inhibitory effect on BChE as compared to the fruits extract (1.70 mg GALAE/g). Regarding tyrosinase inhibition, the leaves extract (50.71 mg KAE/g) had a stronger inhibitory effect than fruit (46.46 mg KAE/g). Like tyrosinase inhibition, the leaves extract (0.53 mmol ACAE/g) was more active on amylase compared to fruit (0.43 mmol ACAE/g). However, the glucosidase inhibition effect of fruit (2.27 mmol ACAE/g) was higher than the leaves extract (2.13 mmol ACAE/g).

**TABLE 4:** Enzyme inhibitory effects of *n*-hexane extracts.

Samples	AChE (mg GALAE/g)	BChE (mg GALAE/g)	Tyrosinase (mg KAE/g)	*α*-amylase (mmol ACAE/g)	*α*-glucosidase (mmol ACAE/g)
Leaves	1.63 ± 0.24	2.24 ± 0.23	50.71 ± 1.12	0.53 ± 0.01	2.13 ± 0.02
Fruits	2.51 ± 0.06	1.70 ± 0.33	46.46 ± 2.19	0.43 ± 0.01	2.27 ± 0.01

Values are reported as mean ± SD, of three parallel experiments. GALAE, galanthamine equivalent; KAE, kojic acid equivalent; ACAE, acarbose equivalent; na: not active. AChE, acetylcholinesterase; BChE: butyrylcholinesterase.

### 3.5 Molecular docking study

The major compounds identified in *n*-hexane fruit and leaves extract of *C. aurantium*, neryl acetate, nootkatone, linalool acetate, decyl anthranilate, friedelan-3-one, lupeol and linalool were docked into the active site vicinity of the five enzymes (i.e., NADPH oxidase, butyrylcholinesterase, tyrosinase, α-amylase, and α-glucosidase). As presented in Table 5, all compounds achieved acceptable binding scores when docked with the five targets.

**TABLE 5:** Docking binding scores of major compounds in *n*-hexane extracts of fruit and leaves of *C. aurantium*.

Tested compounds	2cdu NADPH oxidase,	6esj BChE	5m8q tyrosinase,	4gqq α-amylase	3wy2 α-glucosidase
Neryl Acetate	−8.1	−8.8	−10.9	−8.5	−8.6
Nootkatone	−8.2	−8.6	−8.1	−5.8	−9.7
Linalool acetate	−7.6	−7.3	−11.8	−6.3	−10.4
Decyl anthranilate	−9.7	−8.1	−9.5	−6.4	−10.8
Friedelan-3-one	−11.1	−9.6	−7.1	−6.0	−13
Lupeol	−11.9	−11.4	−8.7	−7.9	−13.2
Linalool	−7.7	−7.4	−6.8	−5.7	−8.6
x-ray References	−7.9	−10.2	−7.6	−7.3	−9.0

BChE, butyrylcholinesterase.

**FIGURE 3:**
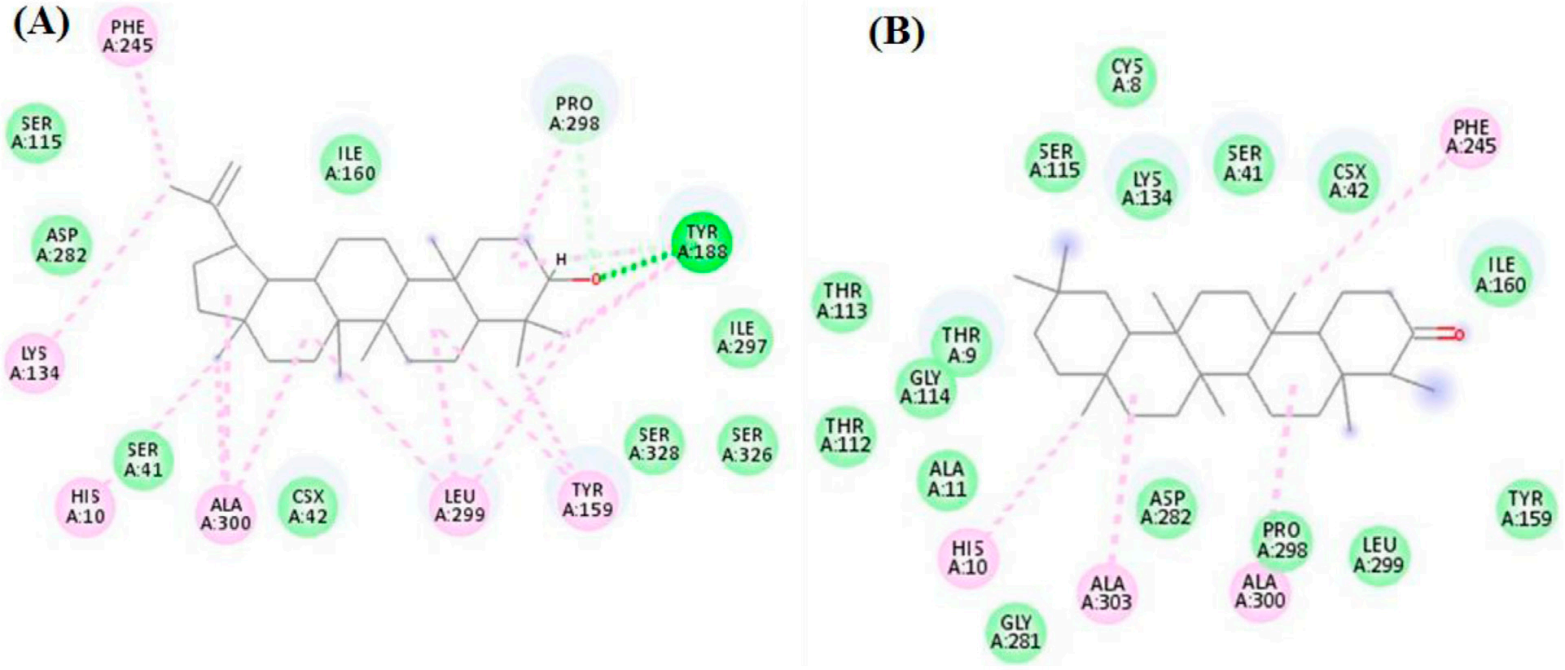
The docking of **(A)** Lupeol **(B)** Friedelan-3-one in the active site of NADPH oxidase enzyme (PDB code: 2cdu).

For the NADPH oxidase, the seven compounds achieved docking scores from −7.6 to −11.9 kcal/mol, where Lupeol and Friedelan-3-one were the best compounds achieving scores of −11.9 and −11.1 kcal/mol, respectively. Inspecting Figure 3, Lupeol interacted with Tyr188 through hydrogen bond interactions and with His10, Lys134, Tyr159, Tyr188, Phe245, Pro298, Leu299 and Ala300 through hydrophobic interactions, while Friedelan-3-one formed only hydrophobic interaction with His10, Phe245, Ala300 and Ala303.

**FIGURE 4:**
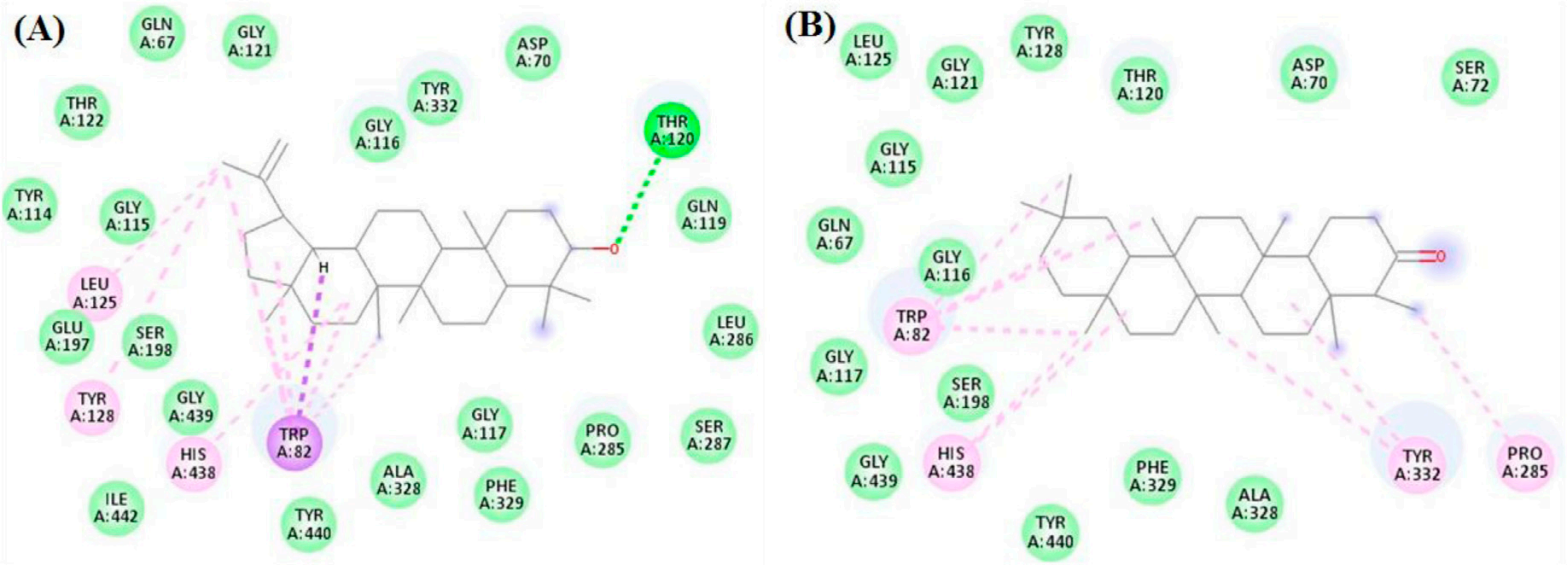
The docking of **(A)** Lupeol, **(B)** Friedelan-3-one in the active site of BChE enzyme (PDB code: 6esj).

For the BChE enzyme, the seven compounds achieved docking scores from −7.3 to −11.4 kcal/mol, where Lupeol and Friedelan-3-one were the best compounds achieving scores of −11.4 and −9.6 kcal/mol, respectively. As seen in Figure 4, Lupeol formed several hydrophobic interactions with Trp82, Leu125, Tyr128, His438, and one hydrogen bond with Thr120. Similarly, Friedelan-3-one formed hydrophobic interactions with Trp82, Pro285, Tyr332, and His438.

**FIGURE 5:**
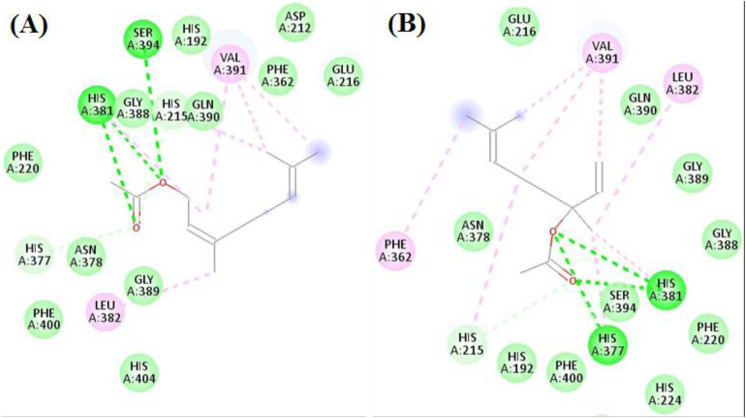
The docking of **(A)** Neryl Acetate, **(B)** Linalool acetate in the active site of tyrosinase enzyme (PDB code: 5m8q).

The seven compounds achieved docking scores ranged from −6.8 to −11.8 kcal/mol against the tyrosinase enzyme. Neryl Acetate and Linalool acetate achieved the best scores −10.9 and −11.8 kcal/mol, respectively. As Figure 5 revealed, Neryl Acetate formed several interactions with His215, His377, His 381, Leu382, Val391, and Ser394. Likewise, Linalool acetate interacted with His215, Phe362, His377, His 381, Leu382, and Val391.

**FIGURE 6:**
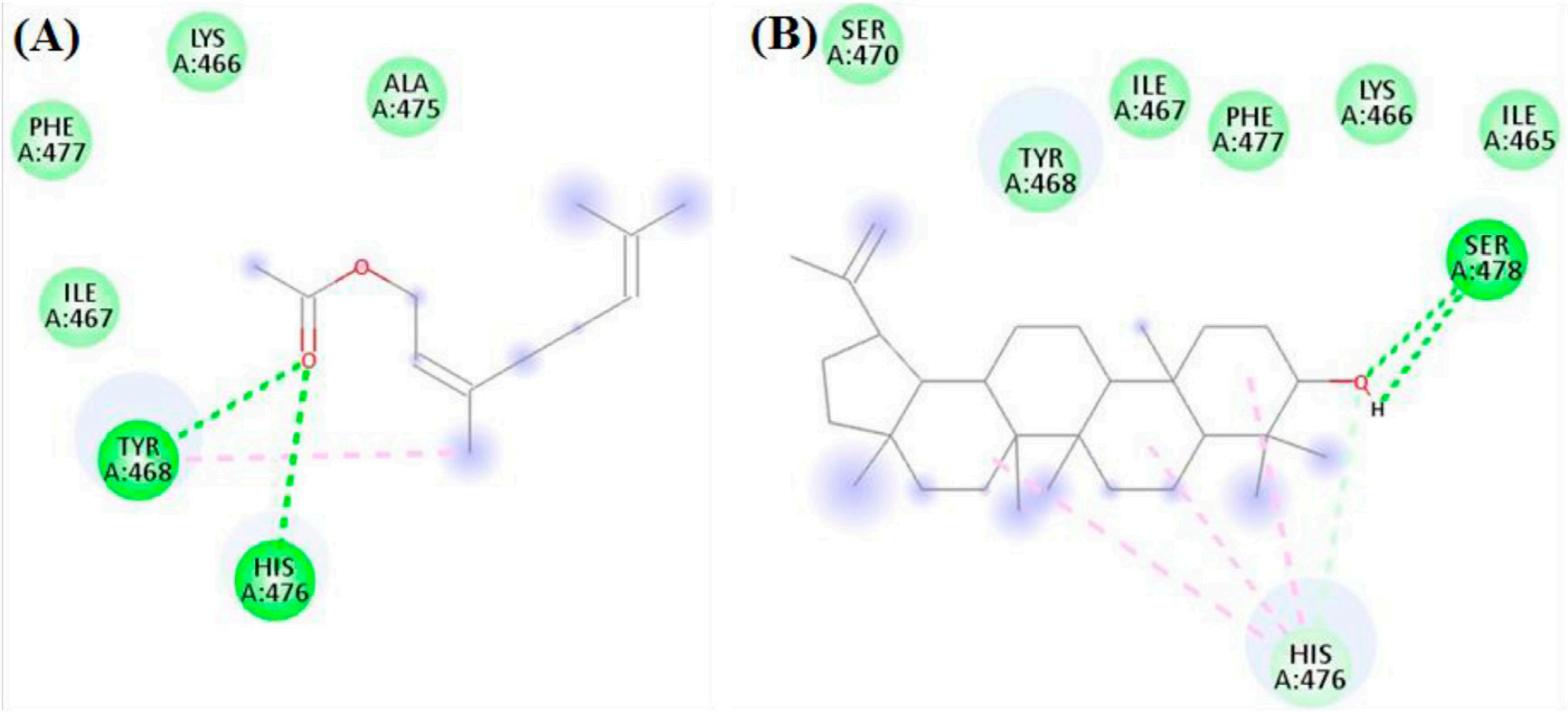
The docking of **(A)** Neryl Acetate, and **(B)** Lupeol, in the active site of amylase enzyme (PDB code: 4gqq).

In the docking with α-amylase, the major compounds achieved good scores ranging from −5.7 to −8.5 kcal/mol. Amongst, Neryl Acetate and Lupeol were the best compounds with scorers −8.5 and −7.9 kcal/mol, respectively. As seen in Figure 6, Neryl Acetate interacted with Tyr468, and His476 through both hydrophobic and hydrogen bond interactions. On the other hand, Lupeol formed both hydrophobic and hydrogen bond interactions with His476 and Ser478, respectively.

For glucosidase, the seven investigated compounds achieved excellent docking scores from −8.6 to −13.2 kcal/mol. Lupeol and Friedelan-3-one achieved docking scores of −13.2 and −13 kcal/mol, respectively, ranking the best two compounds.

**FIGURE 7:**
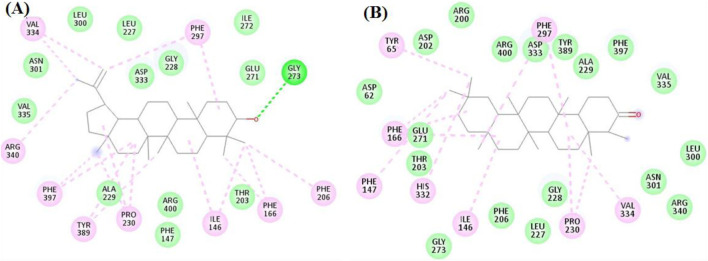
The docking of **(A)** Lupeol, and **(B)** Friedelan-3-one in the active site of glucosidase enzyme (PDB code: 3wy2).

Inspecting their interactions as shown in Figure 7, it was found that Lupeol interacted with, Ile146, Phe166, Phe206, Pro230, Phe297, Val334, Arg340, Tyr389, and Phe397 through hydrophobic interactions, while it formed one hydrogen bond with Gly273. Similarly, Friedelan-3-one interacted with Tyr65, Ile146, Phe147, Phe166, Phe206, Pro230, Phe297, His332 and Val334, through only hydrophobic interactions.

## 4 Discussion

Phenolic compounds are considered multidirectional agents in the development of functional pharmaceuticals and nutraceuticals. Therefore, determining the total phenol content in a plant extract can reveal its biological potential (Mostafa et al., 2018). In this sense, the total bioactive compounds in the extracts were examined and their phenol content was almost the same. However, their total flavonoid content in leaves was almost three times higher than in fruits. In the literature, several authors reported different concentrations of the total bioactive compounds. For example, Lagha-Benamrouche and Madani found that the total phenol content in the leaf extracts of several *Citrus* varieties was between 12.54 and 44.41 mg GAE/g. In another study by Mejri et al., (2023), the total phenolic content of the young leaves of *C. aurantium* was 98.11 mg GAE/g (Mejri et al., 2023). Wang et al. (2023) optimized the extraction of phenolics in the leaves of *C. aurantium* and the level of the total phenolic was 69.09 mg GAE/g in the optimal condition (Wang et al., 2023). In recent years, though, the spectrophotometric results have caused some concern. The results of the tests may be questionable, particularly because the phytochemicals are complex and specific compounds as well as other ingredients may react with the reagent. Further chromatographic techniques are therefore required to validate the results.

The GC-MS of *Citrus aurantium* (*C. aurantium*) extract reveals a rich profile of bioactive compounds, underscores its significant therapeutic potential. This complex chemical composition positions *C. aurantium* as a versatile ingredient for natural health products, with applications in antioxidant, anti-inflammatory, cognitive, and metabolic therapies (Raut and Karuppayil, 2014; Bouabdallah et al., 2022).

The *n-*hexane extract from *C. aurantium* leaves exhibits higher concentrations of linalool and other terpenes that are less prominent in the fruit extract. Linalool, in particular, is associated with sedative, anxiolytic, and anti-inflammatory properties, making leaf extracts particularly valuable for stress relief and inflammation management. The leaves also contain distinctive secondary metabolites not commonly present in the fruit, which contribute to the plant’s defense mechanisms and enhance its therapeutic potential (Chutia et al., 2009).

The chemical composition of *volatile components* can vary significantly depending on the locality, which affects factors like soil composition, climate, season of collection and altitude (Elhawary et al., 2021; Rabie et al., 2023). Comparative studies on *C. aurantium* from Tunisia, Morocco, and other Mediterranean regions highlight variations in the concentration of bioactive compounds in essential oils and extracts, such as limonene, linalool, β-myrcene, α-pinene, and flavonoids. Studies on Tunisian *C. aurantium* essential oils have generally reported high concentrations of limonene and linalool. The Tunisian climate, characterized by a Mediterranean influence with hot summers and mild winters, appears to favor the production of these monoterpenes. For, example, Hosni et al. (2010) found that the essential oils of Tunisian *C. aurantium* are rich in limonene, which can exceed 90% of the volatile fraction. This limonene dominance is associated with strong antioxidant and anti-inflammatory potential, reflecting the suitability of Tunisian *C. aurantium* for applications in antioxidant therapies and skincare products (Karoui and Marzouk, 2013).

In Moroccan *C. aurantium*, linalool and limonene were also major components, yet with slightly different proportions compared to Tunisian sources. Moroccan *C. aurantium* essential oils tend to have a higher proportion of linalool, which is notable for its anxiolytic and sedative effects. This difference is attributed to Morocco’s diverse microclimates, especially in regions with higher altitudes, which seem to enhance the production of compounds with calming properties (Bendaha et al., 2016). Studies have found that Moroccan *C. aurantium* extracts are therefore well-suited for applications in aromatherapy and stress relief products. Egyptian *C. aurantium*, like the samples from Menoufia, Egypt, has a unique profile influenced by the region’s hot and arid conditions. Extracts from the Egyptian chemotype have shown the presence of synephrine, an alkaloid valued for metabolic and stimulant effects, along with limonene and β-myrcene (Ashmawy et al., 2024).

Antioxidants play a protective role against the attacks of free radicals, which trigger the development of serious health problems such as cancer, diabetes or cardiovascular diseases. In addition to health-promoting effects, antioxidants delay lipid peroxidation and thus improve the shelf life of foods in the nutraceutical sector. In this sense, identifying effective and safe sources of antioxidants is one of the most popular topics on the scientific platform. In the study, we determined the antioxidant properties of the tested extracts using *in vitro* methods. In all antioxidant tests, leaf extract was more active than fruit extracts. For example, the ability of leaf extract to intercept DPPH was 2.5 times higher than that of fruit extract. In the literature, several researchers reported significant antioxidant properties of leaves of *C. aurantium* compared to other parts (Mejri et al., 2023; Wang et al., 2023). The antioxidant ability of the leaves extract can be attributed to the presence of some compounds such as lupeol and linalool. For example, Tchimene et al. (2016) isolated lupeol from the leaves of Crateva adansonii and it showed significant capabilities to scavenge DPPH and ABTS as well as an ability to reduce iron (Tchimene et al., 2016).

In addition to DPPH and FRAP, Lupeol had a high capacity to scavenge superoxide, nitric oxide, and hydroxyl radicals, lupeol had great superoxide, nitric oxide and hydroxyl radical scavenging ability (Park et al., 2023). Chaudhary et al. (2023) investigated antioxidant effects of some essential oils and their main components and they reported significant DPPH radical scavenging ability of linalool. Linalool also exhibited significant antioxidant effect in DPPH and FRAP assays. The presence of double bond and hydroxyl group may contribute to the antioxidant effect of linalool.

The theory of enzyme inhibition is currently gaining interest in pharmaceutical applications.

Inhibiting important enzymes can alleviate the symptoms of serious health problems such as diabetes, obesity, or Alzheimer’s. Acetylcholinesterase, for example, is a major target in the treatment of Alzheimer’s disease. Their inhibition led to an increase in acetylcholine levels in the synaptic gap and can therefore improve cognitive functions in Alzheimer patients. Similarly, inhibiting amylase and glucosidase can control blood sugar levels in diabetics, which forms the basis for oral antidiabetic drugs. In this sense, several compounds were produced as enzyme inhibitors, but most of them had adverse side effects. We need therefore to find alternative and safe enzyme inhibitors. Based on these informations, we investigated the enzyme-inhibiting properties of the tested extracts. All extracts inhibited the tested enzymes. The fruit extract showed greater AChE and glucosidase inhibition, but the leaf extract was more active against BChE, tyrosinase, and amylase. In particular, the presence of lupeol in leaf extract can be attributed to enzyme inhibition. Lupeol, as a natural compound with minimal reported toxicity, offers a safer alternative with potentially comparable efficacy, making it a promising candidate for therapeutic development (Park et al., 2023). Lupeol, a naturally occurring triterpenoid, has demonstrated notable inhibitory activity against BChE, an enzyme implicated in neurodegenerative disorders like Alzheimer’s disease. BChE inhibition is particularly significant in late-stage Alzheimer’s, where its activity surpasses acetylcholinesterase (AChE) (Park et al., 2023).

Molecular docking studies have shown that lupeol binds effectively to the active site of BChE, forming stable interactions with key residues, which contributes to its potent inhibitory effect. These interactions involve hydrophobic and hydrogen bonds, enhancing its specificity for BChE over AChE in some cases (Park et al., 2023). Previous studies have reported lupeol’s BChE inhibition with IC50 values in the micromolar range, highlighting its therapeutic potential as a neuroprotective agent. Furthermore, lupeol’s natural origin and low toxicity make it an attractive candidate for drug development targeting cholinesterase-related pathologies (Saleem, 2009). Lupeol has shown significant inhibitory activity against alpha-glucosidase, an enzyme involved in the breakdown of carbohydrates into glucose. Molecular docking and *in vitro* studies suggest that lupeol binds effectively to the active site of alpha-glucosidase, forming hydrogen bonds and hydrophobic interactions with key amino acid residues. These interactions block the enzyme’s catalytic activity, thereby reducing glucose absorption in the intestine. Previous studies have reported that lupeol exhibits alpha-glucosidase inhibition with IC_50_ values in the low micromolar range, comparable to standard antidiabetic drugs like acarbose (Lee et al., 2021). Additionally, its natural origin, combined with antioxidant properties, supports its dual role in glycemic control and protection against oxidative stress associated with diabetes (Lee et al., 2021). In addition to the enzyme-inhibiting effect of lupeol, linalool has been reported as a significant enzyme inhibitor. Alimi et al. (2022) for example, reported a significant AChE inhibitor effect with linalool with an IC50 value of 0.428 mg/mL (Alimi et al., 2022). In addition, López and Pascual-Villalobos (2010) found that linalool competitively inhibited AChE action (López and Pascual-Villalobos, 2010). In the literature, several researchers reported the enzyme-inhibiting potential of members of the genus *Cistus*, including *C. aurantium* (Ashmawy et al., 2024). In this sense, the genus *Cistus* can be considered an effective source of natural enzyme inhibitors.

In the current study, based on the obtained results for the tested biological activities, lupeol achieved the highest docking scores of −11.9, −11.4, and −13.2 kcal/mol for NADPH oxidase, butyrylcholinesterase, and α-glucosidase, respectively. Docking studies of lupeol, a naturally occurring triterpenoid, with NADPH oxidase provide insights into its potential as an inhibitor of oxidative stress pathways (Park et al., 2023). NADPH oxidase is an enzyme complex that produces reactive oxygen species (ROS) as part of cellular signaling and immune response. However, excessive ROS generation due to overactive NADPH oxidase is implicated in various pathological conditions, including cancer, neurodegenerative diseases, and cardiovascular disorders. Lupeol’s interaction with NADPH oxidase could help modulate this enzyme’s activity, offering therapeutic benefits (Park et al., 2023).

In docking studies, lupeol has demonstrated favorable binding affinities within the active or allosteric sites of NADPH oxidase. The molecular structure of lupeol, characterized by its hydrophobic triterpenoid skeleton, allows it to interact effectively with hydrophobic pockets in NADPH oxidase. These interactions may involve hydrogen bonding, hydrophobic interactions, and van der Waals forces with key amino acid residues in NADPH oxidase. Specific residues in the active site, such as those contributing to electron transport, can interact with lupeol, potentially interfering with the enzyme’s ROS-producing function (Chaudhary et al., 2023).

By binding to NADPH oxidase, lupeol may inhibit its activity, thus reducing ROS levels and mitigating oxidative stress. This antioxidant effect could play a role in preventing or slowing the progression of diseases where oxidative damage is a key factor. For instance, in cancer, reducing ROS production could suppress tumor growth and proliferation, as ROS can promote cancer cell survival through multiple pathways (Nakamura and Takada, 2021). Similarly, in neurodegenerative diseases, lowering ROS levels could help protect neurons from oxidative damage, which is central to conditions like Alzheimer’s and Parkinson’s (Nakamura and Takada, 2021).

Lupeol’s docking affinities and binding interactions with NADPH oxidase may be compared to other known NADPH oxidase inhibitors, such as apocynin or diphenyleneiodonium. While synthetic inhibitors often exhibit strong inhibitory effects, they may also produce side effects.

## 5 Conclusion

The current study focused on *Citrus aurantium*, a rich source of bioactive compounds, including nootkatone, decyl anthranilate, neryl acetate, and linalool acetate in fruit hexane extract; and lupeol, linalool, friedelan-3-one and linalool acetate in the leaves extract. The extracts showed *in vitro* bioactivity against AChE, BChE, tyrosinase, amylase, and glucosidase enzymes. These were further confirmed by *in silico* docking studies. The results highlight its therapeutic potential as an antioxidant, neuroprotective, and antidiabetic agent, positioning *Citrus aurantium* as a promising natural source for multi-targeted therapies.

## Data Availability

The original contributions presented in the study are included in the article further inquiries can be directed to the corresponding authors.
